# The transmembrane domain of HIV-1 Vpu is sufficient to confer anti-tetherin activity to SIVcpz and SIVgor Vpu proteins: cytoplasmic determinants of Vpu function

**DOI:** 10.1186/1742-4690-10-32

**Published:** 2013-03-20

**Authors:** Silvia F Kluge, Daniel Sauter, Michael Vogl, Martine Peeters, Yingying Li, Frederic Bibollet-Ruche, Beatrice H Hahn, Frank Kirchhoff

**Affiliations:** 1 Institute of Molecular Virology, Ulm University Medical Center, Ulm, 89081, Germany; 2 UMR 145, Institut de Recherche pour le Développement (IRD), Université de Montpellier, Montpellier, France; 3 Departments of Medicine and Microbiology, University of Pennsylvania, Philadelphia, PA, 19104, USA

## Abstract

**Background:**

The acquisition of effective Vpu-mediated anti-tetherin activity to promote virion release following transmission of SIVcpz*Ptt* from central chimpanzees (*Pan troglodytes troglodytes*) to humans distinguishes pandemic HIV-1 group M strains from non-pandemic group N, O and P viruses and may have been a prerequisite for their global spread. Some functional motifs in the cytoplasmic region of HIV-1 M Vpus proposed to be important for anti-tetherin activity are more frequently found in the Vpu proteins of SIVcpz*Ptt* than in those of SIVcpz*Pts* infecting eastern chimpanzees (*P. t. schweinfurthii),* that have not been detected in humans, and SIVgor from gorillas, which is closely related to HIV-1 O and P. Thus, SIVcpz*Ptt* strains may require fewer adaptive changes in Vpu than SIVcpz*Pts* or SIVgor strains to counteract human tetherin.

**Results:**

To examine whether SIVcpz*Ptt* may only need changes in the transmembrane domain (TMD) of Vpu to acquire anti-tetherin activity, whereas SIVcpz*Pts* and SIVgor may also require changes in the cytoplasmic region, we analyzed chimeras between the TMD of an HIV-1 M Vpu and the cytoplasmic domains of SIVcpz*Ptt* (n = 2), SIVcpz*Pts* (n = 2) and SIVgor (n = 2) Vpu proteins. Unexpectedly, all of these chimeras were capable of counteracting human tetherin to enhance virion release, irrespective of the presence or absence of the putative adaptor protein binding sites and the DSGxxS β-TrCP binding motif reported to be critical for effective anti-tetherin activity of M Vpus. It was also surprising that in three of the six chimeras the gain of anti-tetherin function was associated with a loss of the CD4 degradation activity since this function was conserved among all parental HIV-1, SIVcpz and SIVgor Vpu proteins.

**Conclusions:**

Our results show that changes in the TMD of SIVcpz*Ptt*, SIVcpz*Pts* and SIVgor Vpus are sufficient to render them active against human tetherin. Thus, several previously described domains in the extracellular region of Vpu are not absolutely essential for tetherin antagonism but may be required for other Vpu functions.

## Background

In order to replicate and spread efficiently in their respective hosts primate lentiviruses have to counteract a variety of innate host restriction factors that are frequently induced by type I interferons and inhibit viruses at various steps in their life cycle [1,2]. As a countermeasure, primate lentiviruses have evolved effective antagonists that counteract these restriction factors, e.g. by targeting them for proteasomal degradation or by sequestering them away from their viral targets [3,4]. Both, the antiviral factors and their viral antagonists are under strong selection pressure for diversification [2]. As a consequence, host restriction factors are highly divergent and frequently counteracted by viral factors in a species-specific manner. Thus, they may pose significant hurdles to cross-species transmissions [4].

The genetic barrier for cross-species transmission is reduced between closely related species because pathogens may already be able to evade or counteract some host defense mechanisms. For example, simian immunodeficiency viruses (SIVs) infecting chimpanzees and gorillas that represent the direct precursors of human immunodeficiency virus type 1 (HIV-1) are resistant against human tripartite motif-containing protein 5 (TRIM5α) [5], which induces untimely uncoating of retroviral capsids [6,7]. Furthermore, SIVcpz and (most likely) SIVgor Vif are capable of antagonizing the human APOBEC3G orthologue because it is highly homologous to the corresponding ape proteins [8]. Thus, adaptation of these SIVs to our closest non-human relatives has already disarmed two potent human defense factors. In contrast, primate lentiviruses are unable to counteract the human tetherin orthologue because it contains a deletion in the cytoplasmic region that renders it resistant to the accessory protein Nef, which is used by most SIVs (including SIVcpz and SIVgor) to counteract tetherin [9-11]. As a consequence, tetherin that inhibits virus release by tethering nascent virions at the cell surface seems to constitute a significant barrier to the effective spread of primate lentiviruses in humans [12]. Thus far, only pandemic HIV-1 group M strains have fully cleared this hurdle by the acquisition of effective Vpu-mediated anti-tetherin activity [9]. In comparison, HIV-1 group O and P strains, which resulted from independent zoonotic transmissions and are closely related to SIVgor [13,14] have apparently not yet evolved an effective antagonist of human tetherin [9,15]. Finally, Vpus of the rare HIV-1 N strains acquired some anti-tetherin activity in humans, but lost the second key function of Vpu, i.e. degradation of CD4, the primary viral receptor [9,16].

Molecular epidemiological studies of SIVcpz in wild-living chimpanzees throughout central Africa have shown that only viruses infecting *P.t. troglodytes* but not *P. t. schweinfurthii* apes have crossed the species barrier to humans [17-19], although SIVcpz infection is common in both of them [20]. Furthermore, only the M and N groups of HIV-1 that were transmitted from central chimpanzees *(Ptt)* but not group O and P HIV-1 strains that are most closely related to SIVgor, gained Vpu-mediated anti-tetherin activity during adaptation to humans [9]. The transmembrane domain (TMD) of Vpu seems to interact directly with the TMD domain of tetherin and changes in the TMD were critical for the gain of anti-tetherin activity of group M and N viruses during adaptation to humans [9-11,16]. Furthermore, it has been reported that certain tyrosine- (Yxxϕ) and dileucine-based (E/DxxxLL/I/V/M) sorting motifs in the cytoplasmic domain of Vpu are critical for effective tetherin antagonism by M Vpus [21-24]. Sequence analyses revealed that these motifs are conserved in most SIVcpz*Ptt* Vpus but absent in SIVcpz*Pts* Vpus [20]. Thus, these findings raised the possibility that the Vpu proteins of SIVcpz*Ptt* strains might only have to acquire alterations in the TMD of Vpu to evolve anti-human tetherin activity, whereas those of SIVcpz*Pts* and SIVgor would also need changes in the cytoplasmic region [20]. To examine this possibility, we generated chimeric proteins between the TMD of the HIV-1 M NL4-3 Vpu and the cytoplasmic regions of SIVcpz*Ptt*, SIVcpz*Pts* and SIVgor Vpus. Unexpectedly, we found that all of these chimeric proteins were potent tetherin antagonists, although some of them lack functional domains thought to be important for potent counteraction of tetherin by group M Vpus.

## Results

**Figure 1:**
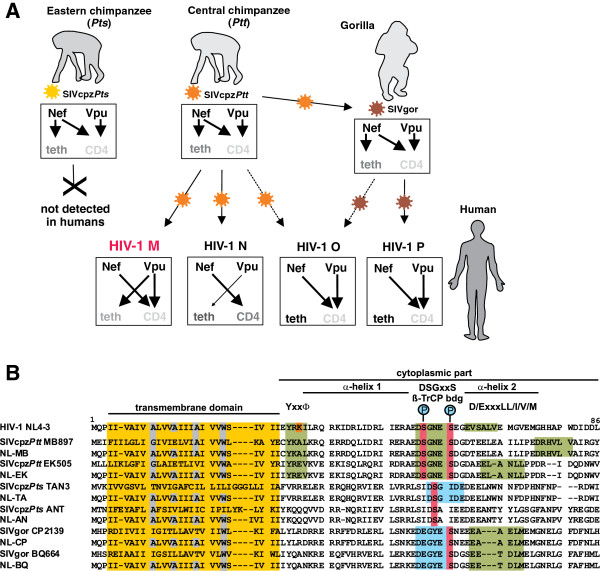
**Zoonotic transmissions of SIVs from apes to humans and adaptive changes in Vpu and Nef functions.** (**A**) The Nef proteins of SIVcpz and SIVgor down-modulate CD4 from the cell surface and counteract tetherin in their non-human hosts. Upon cross-species transmission of SIVcpz and SIVgor to humans, Nef-mediated tetherin antagonism was disrupted by a unique deletion in the cytoplasmic tail of the human tetherin orthologue. Subsequently, Vpu evolved to counteract tetherin during the emergence of pandemic HIV-1 M strains. In contrast, HIV-1 O and P Vpus did not gain anti-tetherin activity. Finally, Vpus of group N viruses evolved some activity against human tetherin but lost their ability to degrade CD4. The arrows indicate activity or cross-species transmissions. Grey indicates antagonism of tetherin or CD4 by one and light grey by two viral factors. (**B**) Alignment of SIVcpz and SIVgor Vpu amino acid sequences. The HIV-1 NL4-3 Vpu sequence is shown on top for comparison. The AxxxAxxxAxxxW residues in the TMD that are important for anti-tetherin activity of M Vpus, a putative Yxxϕ motif, two phosphorylation sites in the DSGxxS ß-TrCP interaction site, and an E/DxxxLL/I/V/M motif involved in targeting of tetherin for endosomal degradation are indicated. The consensus DSGxxS ß-TrCP interaction site is highlighted in green and sites containing mutations of the serines to acidic residues in light blue. Dashes indicate gaps introduced to optimize the alignment.

**Figure 2:**
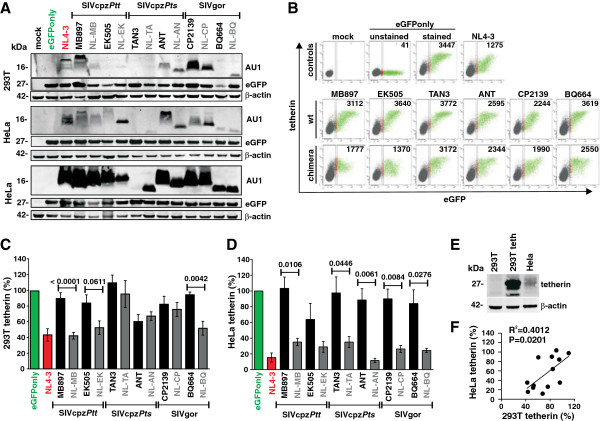
**Down-modulation of human tetherin by chimeras between HIV-1 and SIVcpz/gor Vpus.** (**A**) Expression of wild-type Vpu proteins and chimeras between the TMD of HIV-1 NL4-3 Vpu and the CP of SIVcpz or SIVgor Vpus. 293T cells were transfected with expression plasmids encoding the indicated AU1-tagged Vpus and eGFP. Mock transfected cells were used as negative control. ß-actin and eGFP expression levels were analyzed to control for loading and transfection efficiency, respectively. For HeLa cells two different western blots are shown to allow comparison of Vpu signal intensities (middle) and to show that all Vpus can be detected after long exposure times (bottom). (**B**) FACS analysis of 293T cells co-transfected with a vector expressing tetherin and plasmids expressing eGFP alone or together with the indicated *vpu* allele. The range of eGFP expression levels used to calculate receptor down-modulation and the mean fluorescence intensities (MFI) are indicated. (**C, D**) Vpu-dependent reduction of tetherin surface expression in (**C**) 293T and (**D**) HeLa cells. Shown are the levels of receptor cell surface expression relative to those measured in cells transfected with the eGFP only control vector (100%). Data represent average values (±SEM) derived from three or more experiments. (**E**) Expression of tetherin in untransfected 293T or HeLa cells and in 293T cells transfected with a construct expressing human tetherin (293T teth). (**F**) Correlation between the levels of tetherin expression on 293T and HeLa cells expressing wild-type or chimeric Vpus proteins.

In the present study, we examined whether differential adaptive hurdles of SIVcpz Vpu could explain why only one of two infected subspecies of chimpanzees served as a zoonotic reservoir for humans and why HIV-1 groups O and P that are most closely related to SIVgor [25] failed to acquire Vpu-mediated anti-tetherin activity (Figure 1A). As shown in Figure 1B, several domains reported to be important for the anti-tetherin activity of HIV-1 M Vpus, such as a putative AP-binding Yxxϕ motif [22,26], D/ExxxLL/I/V/M endocytosis motifs [21] and a DSGxxS ß-TrCP interaction domain [26-28], are preserved in the SIVcpz*Ptt* MB897 and EK505 Vpus, but not in those derived from the SIVcpz*Pts* TAN3 and ANT strains. The SIVgor CP2139 and BQ664 Vpus do not contain a Yxxϕ motif adjacent to their TMD and the DSGxxS domain is changed to DEGxxS (Figure 1B). Notably, it has been shown that a phosphorylated serine residue may be mimicked by an acidic residue [29] suggesting that the latter motif may still be capable of interacting with ß-TrCP. To examine whether the SIVcpz*Ptt* Vpus may only require alterations in the TMD to counteract human tetherin, whereas the SIVcpz*Pts* and SIVgor Vpus may need additional changes in cytoplasmic part (CP), we generated a set of chimeras between the TMD of the HIV-1 M NL4-3 Vpu, which is a well characterized antagonist of human tetherin, and the CP of these SIV Vpus (Figure 1B; Additional file 1: Table S1). All wild-type and chimeric Vpus were expressed at detectable albeit highly variable levels in transfected 293T and HeLa cells except for the SIVcpz*Pts* TAN3 Vpu (Figure 2A). The latter was difficult to detect by Western blot, although this protein efficiently degrades CD4 [9]. It is known that the functional activity of Vpu proteins does not always correlate with their *in vitro* expression levels detected by Western blot, possibly because they aggregate and/or remain associated with the insoluble membrane fraction [9,16].

To examine the ability of these wild-type and chimeric Vpu proteins to reduce cell surface expression of tetherin, we transfected 293T cells with constructs co-expressing the various Vpus and eGFP together with a construct expressing human tetherin and analyzed them by flow cytometry. In the absence of Vpu, cells co-expressed high levels of tetherin and eGFP (Figure 2B). In agreement with previous results [9], the NL4-3 Vpu significantly reduced the levels of tetherin expression at the cell surface, whereas the wild-type SIVcpz and SIVgor Vpus were largely inactive (Figure 2B, 2C). The TMD of the NL4-3 Vpu significantly increased the capability of the MB897 and BQ664 Vpus to reduce the surface levels of tetherin in transfected 293T cells, but had only little (if any) effect on the functional activity of the EK505, TAN3, ANT and CP2139 Vpus (Figure 2B, 2C). To test whether the latter was the result of artificial over-expression of tetherin, we examined the of the various wild-type and chimeric Vpu proteins effects on the surface expression of endogenously produced tetherin in HeLa cells (Figure 2D, Additional file 2: Figure S1). We found that the Vpu-dependent reduction of tetherin surface expression was more pronounced in HeLa than in 293T cells, e.g. the NL4-3 Vpu achieved an 85% reduction in the former but only 60% in the latter (Figure 2B-D). This difference in tetherin down-modulation efficiency is most likely a consequence of the higher expression levels in transiently transfected 293T cells (Figure 2E). Unexpectedly, replacement of the SIVcpz and SIVgor Vpu TMD by that of HIV-1 resulted in a reduction of tetherin surface expression in HeLa cells of 60% to 90% (Figure 2D). Even more surprisingly, the chimera between NL4-3 and ANT Vpu (NL-AN) showed the highest activity, although it lacks the Yxxϕ, DSGxxS and E/DxxxLL/I/V/M motifs in the cytoplasmic region (Figure 1B). The results from 293T and HeLa cells were correlated, albeit imperfectly, because some Vpus were substantially more active in the latter cell type (Figure 2F).

**Figure 3:**
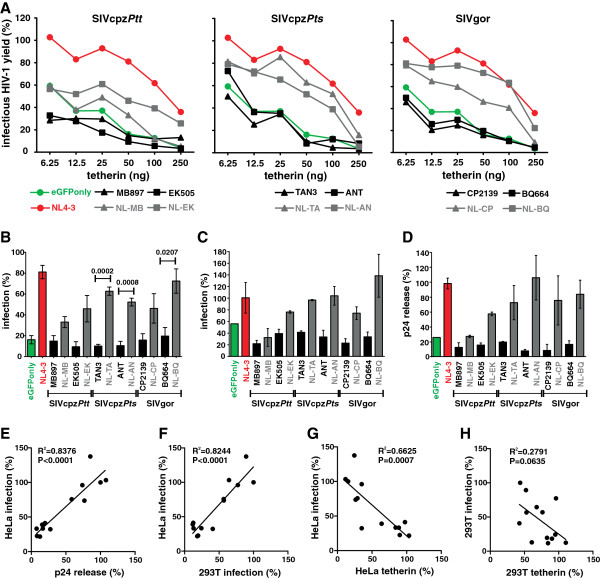
**Enhancement of virus release in the presence of human tetherin by chimeras between HIV-1 and SIVcpz/gor Vpu.** (**A**) Infectious virus yield from 293T cells co-transfected with an HIV-1 NL4-3 Δ*vpu* construct and vectors expressing the indicated *vpu* alleles in combination with various quantities of plasmids expressing tetherin. Shown are average values derived from triplicate infections of TZM-bl indicator cells relative to those obtained in the absence of tetherin (100%). The mean of three independent experiments is shown. The results were verified by measuring the cell-free p24 levels by ELISA. (**B**) Infectious virus yield from 293T cells co-transfected with the proviral HIV-1 NL4-3 Δ*vpu* construct (4 μg), pCGCG vectors expressing the indicated *vpu* alleles (1 ng) and a plasmid expressing human tetherin (50 ng). Shown are average values (±SEM) derived from three independent experiments each involving triplicate infections of TZM-bl indicator cells. (**C, D**) Detection of (**C**) infectious HIV-1 and (**D**) p24 antigen in the supernatant of HeLa cells that express tetherin endogenously and were co-transfected with HIV-1 NL4-3 Δ*vpu* and the indicated Vpu expression constructs. Data represent average values (±SEM) derived from three experiments. (**E-G**) Correlation between infectious virus release from HeLa cells and (**E**) p24 release, (**F**) infectious virus release from 293T cells and (**G**) tetherin cell surface expression levels of HeLa cells. (**H**) Correlation of infectious virus release and tetherin surface expression levels of 293T cells.

To determine the ability of wild-type and chimeric SIVcpz and SIVgor Vpus to promote virus release in the presence of tetherin, we co-transfected 293T cells with a *vpu* defective HIV-1 NL4-3 construct, pCGCG vectors expressing AU1-tagged versions of Vpu and different doses of human tetherin. In contrast to the parental SIVcpz and SIVgor proteins, all chimeras were capable of promoting infectious virus release (Figures 3A). The chimera between the HIV-1 TMD and the SIVgor BQ664 CP (NL-BQ) increased infectious virus release in both 293T and HeLa cells almost as efficiently as the control NL4-3 Vpu and was substantially more effective than the chimeras between HIV-1 and SIVcpz*Ptt* Vpus (NL-MB, NL-EK) (Figures 3A, 3B). This was surprising because in contrast to the SIVcpz*Ptt* MB897 and EK505 Vpus, the cytoplasmic part of the SIVgor BQ664 Vpu contains substitutions in the DSGxxS motif (DEGYES) and lacks a putative Yxxϕ motif (Figure 1B). These results were confirmed by measuring the quantity of fectious virus and p24 release in the culture supernatant of HeLa cells co-transfected with proviral HIV-1 and Vpu expression constructs (Figures 3C to E). All Vpus that promoted infectious virus release in transiently transfected 293T cells were also active in HeLa cells (Figure 3F) and most Vpu proteins that reduced tetherin surface expression also enhanced virion release (Figure 3G, 3H).

**Figure 4:**
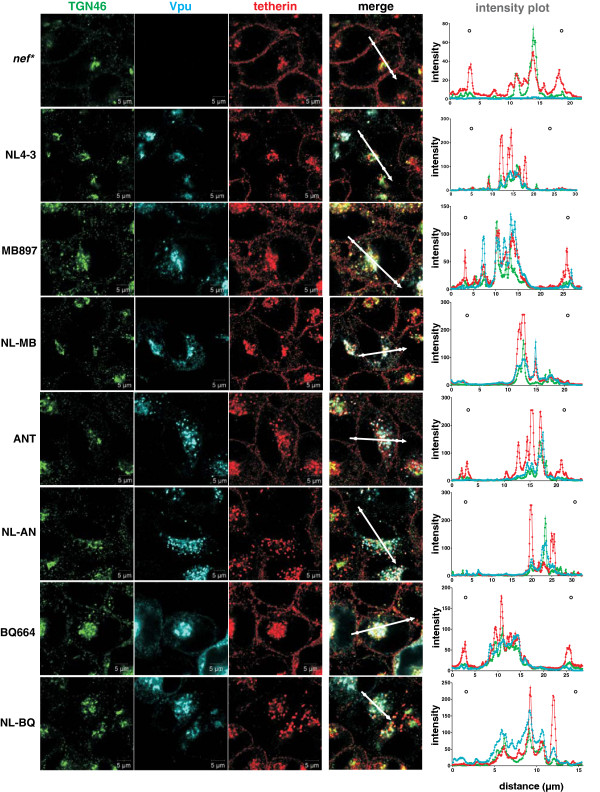
**Cellular localization of tetherin in the presence of various Vpu proteins.** Confocal immunofluorescence images of HeLa cells transfected with constructs expressing the indicated Vpu variants. Two days post-transfection, cells were fixed and permeabilized for intracellular staining of tetherin (red), Vpu (blue) and the TGN (green). Images show representative confocal acquisitions of at least 40 transfected cells investigated. Tetherin was located at the cell surface in cells transfected with constructs expressing the parental SIVcpz and SIVgor Vpus but not in cells expressing chimeras containing the TMD of the NL4-3 Vpu. Cellular localization was determined by microscopic examination and by analysis of the Vpu, tetherin and TGN signal intensities throughout the cells (right panel). The regions utilized to generate the profile plots are indicated by the arrows. White circles indicate the position of the plasma membrane.

To further analyze the unexpected activity of the chimeric Vpus against human tetherin, we next performed microscopic analyses in HeLa cells to examine their effect on the subcellular localization of this restriction factor. In the absence of Vpu and in cells expressing wild-type SIVcpz*Ptt*, SIVcpz*Pts* and SIVgor Vpu proteins, tetherin co-localized with the trans-Golgi network (TGN) marker TGN46 but was also present at the cell surface (Figure 4). In contrast, all Vpu chimeras prevented tetherin expression at the cell surface as effectively as the wild-type NL4-3 Vpu (Figure 4). Thus, the results of the microscopic analyses confirmed that the HIV-1 TMD SIVcpz/gor CP chimeras are effective tetherin antagonists that promote effective virion release by sequestering the restriction factor away from the cell surface. Our results further suggest that co-localization of Vpu and tetherin in the TGN may be required but not sufficient for tetherin antagonism.

**Figure 5:**
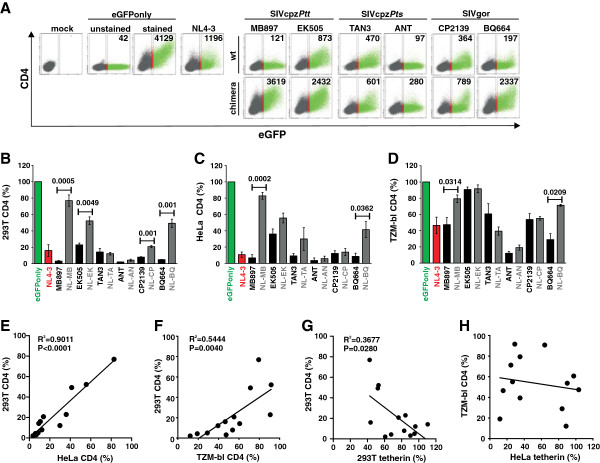
**Modulation of CD4 by chimeras between HIV-1 and SIVcpz/gor Vpus. (A)** FACS analysis of 293T cells co-transfected with a CD4 expression vector and pCGCG plasmids expressing eGFP alone (eGFPonly) or together with the indicated *vpu* alleles. A construct expressing NL4-3 Vpu was used as a positive control. (**B-D**) Levels of CD4 surface expression in (**B**) 293T, (**C**) HeLa and (**D**) TZM-bl cells in the presence of the indicated *vpu* alleles are compared to those measured in the absence of Vpu (100%). Values give averages (±SEM) derived from three to five independent experiments. The same ranges of eGFP expression were used in all calculations. (**E, F**) Correlation between CD4 cell surface levels on 293T and (**E**) HeLa or (**F**) TZM-bl cells. (**G, H**) Correlation between CD4 and tetherin cell surface levels on (**G**) 293T cells and (**H**) TZM-bl and HeLa cells.

Another well established function that is conserved among all primate lentiviral Vpus, except for those of HIV-1 group N strains [9,16], is the ability to degrade the CD4 receptor [30-32]. To determine whether the Vpu chimeras were active against CD4, we co-transfected 293T cells with vectors co-expressing Vpu and eGFP (or eGFP alone for control) together with a CD4 expression construct. In the absence of Vpu, cells expressed high levels of CD4 and eGFP (Figure 5A, Additional file 3: Figure S2). All wild-type HIV-1, SIVcpz and SIVgor Vpu proteins markedly reduced CD4 cell surface expression, whereas several Vpu chimeras were poorly active (Figure 5A). Quantitative analyses showed that the parental Vpus usually reduced CD4 cell surface expression by more than 80%, whereas the NL-MB, NL-EK and NL-BQ chimeras only achieved 20% to 50% reduction (Figure 5B). Notably, the chimeras between the HIV-1 Vpu TMD and the SIVcpz*Pts* TAN3 and ANT as well as SIVgor CP2139 CPs of Vpu maintained most of their anti-CD4 activity (Figure 5B). Analysis of HeLa cells transfected with CD4 expression constructs and of TZM-bl cells stably expressing CD4 (Additional file 3: Figure S2) confirmed these results (Figure 5C-F). The effects were generally weaker in TZM-bl cells than in transiently transfected 293T and HeLa cells (Figure 5B-D). This is most likely due to the fact that Vpu mediates efficient degradation of intracellular CD4 but is unable to remove CD4 molecules from the surface that were already at the plasma membrane prior to transfection [27,30-32]. In 293T cells, we observed a significant inverse correlation between the effect of the panel of Vpu protein analyzed on tetherin and CD4 surface expression (Figure 5G). The same trend was found in HeLa cells but failed to reach significance (Figure 5H) because the NL-AN and NL-CP chimeras reduced the surface expression of both receptors (Figures 2D and 5C). These results suggest that the acquisition of changes in the TMD of Vpu that confer anti-tetherin activity may have detrimental effects on the CD4 degradation activity in the context of some SIVcpz and SIVgor *vpu* alleles.

**Figure 6:**
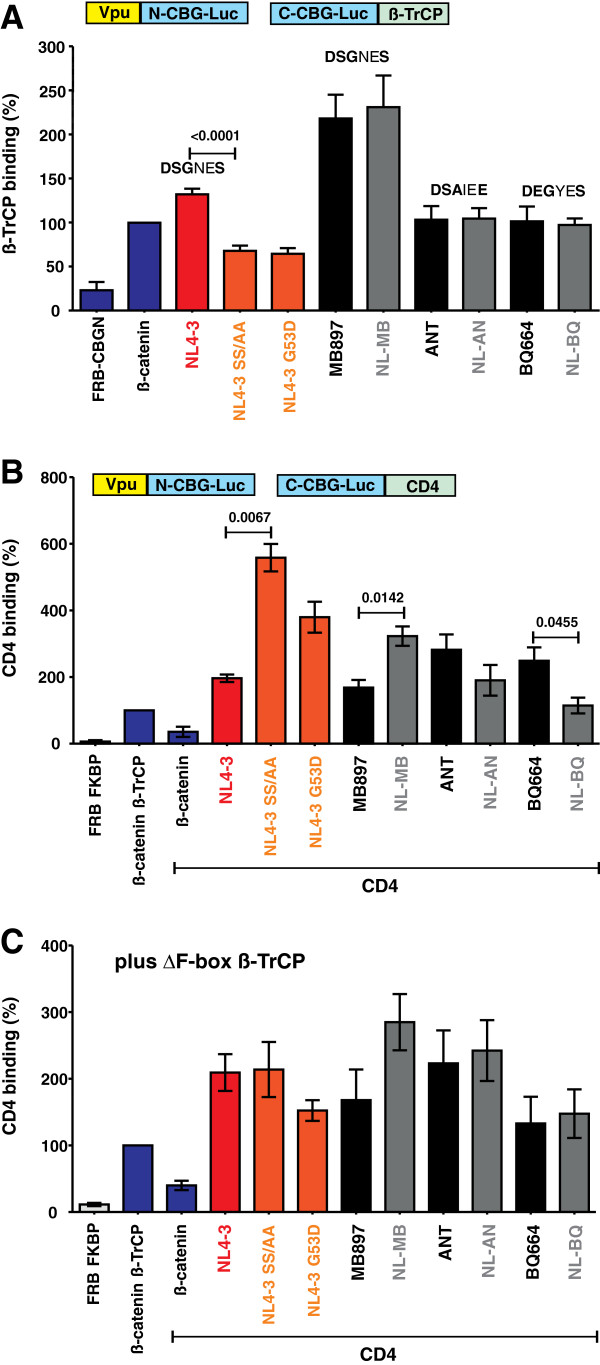
**Interaction of chimeras between HIV-1 and SIVcpz/gor Vpus with ß-TrCP and CD4.** (**A**) Interaction of Vpu with ß-TrCP. 293T cells were transfected with equal amounts of plasmids expressing ß-TrCP N-terminally fused to the C-terminal fragment of click beetle green (CBG) and Vpu C-terminally fused to the N-terminal fragment of CBG. ß-catenin served as positive control. After 48 h, click beetle luciferase activity was determined in living cells by addition of D-luciferin and quantification of bioluminescence. Values represent means (±SEM) derived from five independent experiments. (**B**) Interaction of Vpu with CD4. 293T cells were transfected with plasmids expressing CD4 C-terminally fused to the C-terminal fragment of CBG and Vpu C-terminally fused to the N-terminal fragment of CBG and interaction efficiencies were determined as described in the methods section. (**C**) To exclude a bias caused by Vpu-mediated degradation of CD4, a dominant negative mutant of β-TrCP1 isoform 2 lacking the F-box (amino acids 141–193) was co-transfected in a control experiment. This mutant still binds Vpu but lacks the Fbox domain and thus fails to recruit the E3 ubiquitin ligase complex. Values represent means (±SEM) derived from three to four independent experiments.

Vpu interacts with the cytoplasmic part of CD4 and binds ß-TrCP to recruit an E3 ubiquitin ligase complex to CD4 thereby inducing its degradation by cellular proteasomes [27]. Effective CD4 degradation by M Vpus requires a functional DSGxxS β-TrCP target motif, where the serines need to be phosphorylated to allow efficient recruitment of β-TrCP [31,32]. We have previously shown that SIVcpz and SIVgor Vpus are highly active against CD4 [9], although the DSGxxS motif is frequently altered (e.g. to DSAIEE or DEGYES) and contains only a single putative phosphorylation site (Figure 1B). To examine whether the wild-type and chimeric Vpus are capable of interacting with ß-TrCP, we fused the N-terminal fragment of the click beetle luciferase to the C-terminus of Vpu, and the C-terminal fragment of this luciferase to the N-terminus of ß-TrCP (Figure 6A). If Vpu and ß-TrCP interact, functional luciferase is assembled [33]. The interaction of ß-catenin and ß-TrCP served as positive control [33]. We found that the wild-type HIV-1 and SIVcpz or SIVgor Vpus interacted with ß-TrCP, although the detectable levels of luciferase reporter activity varied (Figures 6A, Additional file 4: Figure S3A). In agreement with published data [28,32,34,35], mutations in the DSGxxS motif of the NL4-3 Vpu (S52A/S56A or G53D) significantly reduced this interaction. The ANT and BQ664 Vpus that contained alterations in the β-TrCP binding site showed weaker interaction than the MB897 Vpu (Figure 6A, Additional file 4: Figure S3A), which has the consensus DSGxxS motif. However, all of them were highly effective in reducing CD4 cell surface expression (Figure 5). Most importantly, exchanges of the TMD had no significant effect on the interaction of Vpu with ß-TrCP (Figure 6A, Additional file 4: Figure S3A). Thus, reduced ß-TrCP binding was not the reason for the reduced anti-CD4 activity of some chimeric Vpus.

Next, we used the click beetle complementation assay to examine whether replacement of the TMD of SIVcpz or SIVgor Vpus may affect their ability to bind CD4. We found that all Vpus resulted in increased (albeit variable) levels of luciferase reporter activity compared to ß-catenin, which has not been shown to interact with CD4. This suggests that all Vpus are capable of interacting with CD4 (Figure 6B, Additional file 4: Figure S3B). Mutations in the ß-TrCP binding site of the NL4-3 Vpu increased the signal intensities because the binding of ß-TrCP and subsequently CD4 luciferase reporter construct degradation was impaired (Figure 6B, Additional file 4: Figure S3B). Replacement of the TMDs of the MB897, ANT and BQ664 Vpus by that of the NL4-3 Vpu had differential and modest effects on luciferase activity. To examine whether some of these differences were the result of an altered interaction or Vpu-mediated degradation of the CD4 luciferase reporter construct, we repeated the interaction assay in the presence of a *trans*-dominant negative mutant of ß-TrCP1 (TrCP1ΔFbox). This mutant still binds Vpu via WD repeats but lacks the Fbox, and thus fails to recruit the E3 ubiquitin ligase complex for proteasomal degradation of CD4 [32]. In the presence of the TrCP1ΔFbox mutant, all Vpu chimeras resulted in similar or moderately increased (NL-MB) levels of luciferase reporter activities compared to the respective wild-type SIVcpz and SIVgor Vpus proteins (Figure 6C, S3C) suggesting that the exchange of the TMD motif did not disrupt CD4 binding. Thus, the NL-MB and NL-BQ Vpu chimeras were poorly active in reducing CD4 cell surface expression (Figure 5), although they were efficiently expressed (Figure 2A) and capable of interacting with both ß-TrCP and CD4 (Figure 6, Additional file 4: Figure S3).

## Discussion

In the present study, we show that the Vpu proteins of six SIVcpz*Ptt*, SIVcpz*Pts* and SIVgor strains became antagonists of human tetherin when their TMD was replaced by that of the HIV-1 M NL4-3 Vpu. The TMD domain of HIV-1 Vpu interacts with human tetherin [24,34,36] and recent data suggest that this interaction is sufficient for some anti-tetherin activity [16,21,26]. Efficient counteraction of tetherin by M Vpus, however, also requires several cytoplasmic domains [21,22,26,28,35]. It has been suggested that the absence of these motifs contributes to the lack of anti-tetherin activity of O Vpus [26] and may explain the poor activity of group N Vpus against human tetherin [16]. The Vpu proteins of many SIVcpz and SIVgor strains, in particular those from SIVcpz*Pts* strains, lack the canonical functional motifs. Thus, it came as a surprise when the majority of chimeric Vpus were almost as effective in reducing cell surface expression of tetherin and in promoting virus release as the group M NL4-3 Vpu. Even more strikingly, the most active chimeras were not the ones between HIV-1 and SIVcpz*Ptt* Vpus that contained the canonical Yxxϕ, E/DxxxLL/I/V/M and DSGxxS β-TrCP binding motifs, but those between HIV-1 and SIVcpz*Pts* or SIVgor Vpus that lacked most or all of these putative domains [20] (Figure 1B). These results show that the presence or absence of “binding motifs” important for effective tetherin antagonism by M Vpus cannot be used to predict the functionality of the cytoplasmic domain of SIVcpz and SIVgor Vpus. The data also suggest that most SIVcpz and SIVgor Vpus, regardless of their genetic diversity, require only changes in their TMD to become active against human tetherin.

Since SIVcpz and SIVgor clearly use Nef to counteract the tetherin restriction in their natural ape hosts [9], it may seem striking that the cytoplasmic domains of their Vpu proteins fulfill the requirements to antagonize tetherin. The *vpu* gene is believed to have originated in a precursor of SIVs that infects guenons (SIVgsn/mus/mon), which subsequently recombined with an SIV strain that infects red-capped mangabeys (SIVrcm) to generate SIVcpz [37,38]. The Vpus from current SIVgsn, SIVmus and SIVmon strains are all capable of counteracting the tetherin orthologue of their cognate host species [9]. Thus, the Vpu that SIVcpz received from the precursor of the SIVgsn/mus/mon lineage was most likely a functional tetherin antagonist. However, due to sequence variations in the transmembrane portion of Vpu, tetherin is counteracted in a species-specific manner and subsequently Nef and not Vpu evolved to become an effective tetherin antagonist in chimpanzees [9]. It is tempting to speculate that the cytoplasmic domain of Vpu maintained the requirements for counteracting tetherin because the sequence motifs in Vpu that enable proper subcellular localization and/or are involved in the recruitment of the ubiquitination/degradation machinery are also critical for CD4 degradation and (possibly) for other Vpu activities, such as down-modulation of the natural killer cell ligand NTB-A and the lipid-antigen presenting protein CD1d [39-41]. It is conceivable that domains that are beneficial for several functions will be preserved even if one function is lost. Indeed, recent data have shown that just two amino acid changes are sufficient to render HIV-1 Nef active against chimpanzee tetherin, suggesting that other functional constraints keep Nef “ready” to regain this function [42].

The results of the present study further illustrate the functional plasticity of the viral accessory proteins. It seems clear that several domains that may be critical for full activity of M Vpus are not required for effective tetherin antagonism by chimeric Vpu proteins. Thus, the functional relevance of some cytoplasmic domains in Vpu seems to be context dependent. Similarly, we have previously observed that some SIV or HIV-2 Nef proteins are fully functional, although they lack some of the domains reported to be critical for HIV-1 Nef function [43,44]. It is often difficult to assess whether the required functional interactions with cellular factors are performed by different regions of the viral proteins or whether the sequence variations are tolerated such that the mutated domains remain active. For example, it has been reported that phosphorylation of both serines in the HIV-1 DSGxxS domain is essential for Vpu-mediated degradation of CD4 [45]. However, a phosphorylated serine residue may be mimicked by an acidic residue [29]. For example, SIVgor Vpus that contain a DEGxxS domain interact with ß-TrCP and downregulate CD4 [9]. Furthermore, some Vpu proteins that do not contain the Yxxϕ and E/DxxxLL/I/V/M motifs in their cytoplasmic region may use different domains to interact with cellular trafficking or endocytosis motifs. For example, a NPxY (or FxNPxY or FxNPxF) motif is found in many SIVcpz and most HIV-1 O Vpus. It is known that this tyrosine motif allows other type I transmembrane proteins (e.g. the LDL and insulin receptors) to recruit AP-2 [46-48], but its relevance for Vpu function remains to be investigated. In either case, our results indicate that the presence or absence of specific sequence motifs in lentiviral accessory proteins do not necessarily predict their functional integrity.

Our results show that it is difficult to predict the changes required for a gain of anti-tetherin activity of SIVcpz or SIVgor Vpus based on results obtained using M Vpus because the effect of sequence alterations is often context dependent. While our data indicate that SIVcpz*Ptt*, SIVcpz*Pts* and SIVgor Vpus may require mainly mutations in their TMDs to acquire significant anti-tetherin activity, they do not allow to estimate which and how many amino acid substitutions are required for optimal adaptation. For example, the adaptive changes in the TMD that allow HIV-1 group M and N Vpus are overlapping, but distinct [16,49]. Thus, our findings do not refute the previously suggested possibility that SIVcpz*Ptt* may on average require fewer adaptive changes than SIVcpz*Pts* strains to become functional in humans [20].

To avoid possible artifacts due to over expression of tetherin, we examined the effects of Vpu in both transiently transfected 293T cells and in HeLa cells, which express tetherin endogenously. In HeLa cells, the effects of the various wild-type and chimeric Vpus on the reduction of tetherin cell surface expression and virus release correlated significantly (Figure 3G). However, the effects of Vpu on cell surface expression of tetherin were weaker in 293T cells and some Vpu chimeras clearly reduced tetherin surface expression in HeLa cells, but failed to exert a significant effect in 293T cells (Figure 2C, 2D). The reasons for these discrepancies remain unknown, but may involve higher expression levels (Figure 2E) as well as different glycosylation patterns of tetherin. Nonetheless, these results suggest that potent anti-tetherin effects can be monitored in 293T cell-based over-expression assays, but weaker activities against tetherin may be missed.

In some cases, a gain of anti-tetherin activity by the Vpu chimeras was associated with a significant loss of the CD4 degradation function. This came as a surprise because all parental Vpus were active against CD4 and the chimeras thus contained all functional domains required for this activity. The inverse correlation between both functions was dependent on the context of the specific SIVcpz or SIVgor *vpu* alleles utilized to generate the Vpu chimeras. Specifically, chimeras between the TMD of HIV-1 Vpu and the CP of SIVcpz*Ptt* MB897 and EK505 or SIVgor BQ664 Vpu (NL-MB, NL-EK, NL-BQ) were poorly active against CD4, whereas the remaining three chimeras (NL-TA, NL-AN and NL-CP) potently suppressed CD4 cell surface expression (Figure 5). Thus, the acquisition of TMD changes facilitating tetherin interaction and counteraction can come at a cost, i.e., the loss in the CD4 degradation function. It remains a matter of speculation whether this may explain the loss of the CD4 degradation activity of group N Vpus [9]. The reasons for the loss of CD4 down-regulation function in some Vpu chimeras also remain to be determined as they contain all known interaction domains and bind both CD4 and ß-TrCP molecules (Figure 6). Furthermore, efficient expression and anti-tetherin activity of these Vpu chimeras argue against major structural defects. It thus seems clear that the TMD and CP of Vpu must interact in some way for full functionality of the protein.

## Conclusions

A better understanding of the adaptive barriers that primate lentiviruses have to overcome to spread efficiently in humans is essential to assess the potential risk of future pandemics and may help to develop new preventive strategies. Previous results suggest that effective tetherin antagonism may have been a prerequisite for the effective spread of pandemic HIV-1 M strains [12,50] and that easier adaptability of Vpu to humans may explain why only certain SIVcpz and SIVgor strains have become new human pathogens [20]. We show here that Vpus from all major SIVcpz and SIVgor lineages require only changes in their TMD to acquire activity against human tetherin. Thus, these ape viruses seem to be more prone to acquire anti-tetherin activity in humans than previously anticipated and it is thus surprisingly that group O and N Vpus have not yet evolved anti-tetherin activity during adaptation to humans. One possible reason for this is that these changes may compromise the CD4 degradation function of Vpu, which may help to explain the loss of the latter activity during adaptation of HIV-1 group N to humans. Our results illustrate that the plasticity and multi-functionality of primate lentiviral accessory proteins makes reliable predictions concerning their zoonotic potential difficult. Notably, the same multi-functionality may also allow primate lentiviruses to acquire new antagonisms, because functions for endocytosis or proteasomal degradation are already in place.

## Methods

### Expression vectors and proviral constructs

Bi-cistronic CMV-promoter-based pCGCG vectors co-expressing Vpu or tetherin and the enhanced green fluorescent protein (eGFP) or dsRed2, respectively, have been described previously [9]. The *vpu* alleles were not codon optimized. Splice-overlap-extension PCR was used to introduce an AU1-tag as well as *XbaI* and *MluI* restriction sites flanking the reading frames. The PCR fragments were cloned into the pCGCG vector using standard cloning techniques. To inhibit β-TrCP mediated recruiting of the E3 ubiquitin ligase to Vpu, a dominant negative mutant of β-TrCP1 isoform 2 lacking the Fbox (amino acids 141–193) was synthesized (Genscript, Piscataway, USA) and cloned into the pCGCG vector co-expressing dsRed2 via an IRES [16]. All PCR-derived inserts were sequence confirmed. A *vpu* deleted mutant of a CCR5-tropic HIV-1 NL4-3 proviral derivative [51] was used to determine the effect of various Vpus on infectious virus release following complementation *in trans*.

### Cell culture

293T and HeLa cells were maintained in Dulbecco modified Eagle medium (DMEM) supplemented with 10% heat-inactivated fetal bovine serum, 350 μg/ml L-glutamine, 120 μg/ml streptomycin sulfate and 120 μg/ml penicillin. TZM-bl cells, a HeLa cell line derivative that expresses large amounts of CD4, CCR5 and CXCR4 and contain the ß-galactosidase gene under the control of the HIV-1 promoter [52-54] were kindly provided by Drs. Kappes and Wu and Tranzyme Inc. through the NIH AIDS Reagent Program and were kept in DMEM supplemented as described above.

### Western blot

To monitor Vpu expression, HeLa cells were transfected with 5 μg of the expression constructs. Two days post-transfection cells were harvested, lysed in M-PER buffer (ThermoScientific) containing 1% SDS and a protease inhibitor cocktail (Roche) and cell lysates were separated in 10% SDS-PAA gels in a Tris-Tricine buffer system. After gel electrophoresis, proteins were transferred onto PVDF membranes and probed with anti-AU1 antibody (Covance, MMS-130P). For internal controls, blots were incubated with antibodies specific for eGFP (290–50, Abcam) and β-actin (8227–50, Abcam). Subsequently, blots were probed with anti-mouse or anti-rabbit IRDye Odyssey antibodies (926–32210, 926–32221) and proteins detected using a LI-COR Odyssey scanner.

### Flow cytometric analysis

To determine the effect of Vpu on tetherin and CD4 cell surface expression, 293T were transfected in 6-well plates by the calcium phosphate method as described previously [55] and HeLa cells using Lipofectamine LTX reagent (Invitrogen) with 1 μg of a tetherin (for 293T cells) or CD4 (for 293T and HeLa cells) expression vector and 5 μg of pCGCG constructs expressing eGFP alone (as control) or together with Vpu. Two days post-transfection, tetherin or CD4 expression was examined by FACS analysis, as described previously [56]. An allophycocyanin (APC)-conjugated anti-human CD4 antibody (Invitrogen; MHCD0405) was used for staining of surface CD4. For staining of surface tetherin an unconjugated anti-tetherin antibody (eBioscience) and an APC-conjugated secondary anti-mouse antibody (Invitrogen, A865) were used. Fluorescence of stained cells was detected by two-color flow cytometry and Vpu-mediated tetherin or CD4 down-modulation was calculated as described previously for the functional analysis of *nef* alleles [37]. Briefly, for exogenously expressed proteins, the mean fluorescence intensity (MFI) obtained for cells transfected with the control construct expressing only eGFP was compared to the MFI obtained for cells co-expressing Vpu and eGFP to determine the efficiency of tetherin or CD4 down-regulation. For endogenously expressed proteins, the MFI of untransfected cells (eGFP negative) was compared to the MFI of transfected cells (eGFP positive). The transfection of the control construct expressing only eGFP served as reference.

### Virus release assays

To determine the capability of Vpu to antagonize tetherin, 293T or HeLa cells were seeded in six-well plates and transfected with 4 μg of NL4-3 Δ*vpu* IRES eGFP, 1 μg Vpu expression plasmid and (for 293T cells) different amounts of a tetherin expression vector (6.25, 12.5, 25, 50, 125 and 250 ng). Two days post-transfection, supernatants were harvested and analyzed for infectious virus release by a 96-well infection assay on TZM-bl indicator cells [55] and the release of p24 antigen by ELISA as described previously [9].

### Microscopy

HeLa cells were transfected using Lipofectamine LTX Reagent (invitrogen) with pCGCG Vpu_AU1 constructs and stained 24 hours post-transfection. Briefly, cells were fixed with 4% PFA, blocked with BSA and surface tetherin was stained with an anti-tetherin antibody (eBioscience) and a secondary antibody conjugated to Alexa Fluor® 647 (Invitrogen). Cells were then permeabilized with 0.5% saponin. After blocking with BSA, Vpu, tetherin and TGN46 were stained with an anti-AU1 (Covance), anti-tetherin (eBioscience) and anti-TGN46 (Serotec) antibody, respectively. Secondary antibodies conjugated to Alexa Fluor® 568, Alexa Fluor® 647 and Alexa Fluor® 488 were used for detection (Invitrogen). A confocal microscope (LSM 710, Zeiss) with the corresponding software (Zeiss Zen Software, 2010) was used for analysis.

### Click beetle assay

The click beetle luciferase heteroprotein fragment complementation assay allows the real-time analysis of protein-protein interactions in living cells [33]. pCBG-C_ß-TrCP1 and pß-catenin_CBG-N constructs encoding the N- or C-terminal fragment of click beetle green (CBG) were kindly provided by Piwnica-Worms. SalI and BamHI restriction sites were added to the vpu alleles by PCR and standard cloning techniques were used to insert the Vpu alleles into the pCBG-C vector replacing the ß-catenin gene. Splicing by overlap extension PCR (SOE-PCR) was used to fuse the C-terminal CBG-fragment to the C-terminus of human CD4. The ß-TrCP1_CBG-C gene was replaced by the CD4_CBG-C fusion gene via HindIII and XbaI digestion. The luciferase assay was essentially performed as described before [33]. Briefly, 293T cells in white 96-well plates with clear bottom were transfected with equal amounts of the click beetle green constructs. 48 hours after transfection the cells were washed once and then incubated in MEBSS buffer (Modified Eagle’s balanced salt solution: 144 mM NaCl, 5.4 mM KCl, 0.8 mM MgSO_4_, 0.8 mM NaH_2_PO_4_, 1.2 mM CaCl_2_, glucose 5.6 mM, and HEPES 4 mM [pH 7.4]) containing 1% heat-inactivated fetal bovine serum and 150 μg/ml D-Luciferin. Photon flux was quantified at room temperature with a SAFAS Xenius spectrofluorimeter for 1 min or an Orion microplate luminometer for 10 sec.

### Sequence analysis

Vpu amino acid sequences were aligned using multiple sequence alignment with hierarchical clustering (http://multalin.toulouse.inra.fr/multalin/). Vpu sequences were obtained from the HIV Sequence Database (http://www.hiv.lanl.gov).

### Statistical methods

The activities of vpu alleles were compared using a two-tailed Student’s *t* test. The PRISM package version 4.0 (Abacus Concepts, Berkeley, CA) was used for all calculations.

## Competing interests

The authors declare that they have no competing interest.

## Authors’ contributions

SFK performed the experiments. MV, MP, YL, FBR and BHH contributed reagents. DS participated in the coordination of the study and in the review of the manuscript. FK designed and coordinated the study and wrote the final manuscript together with BHH. All authors have read and approved the final manuscript.

## Supplementary Material

Additional file 1: Table S1 Origin of HIV-1, SIVcpz and SIVgor vpu alleles analyzed. Click here for file

Additional file 2: Figure S1 Tetherin (upper panel) or CD4 (lower panel) surface expression levels of HeLa cells co-transfected with a CD4 (lower panel) expression vector and pCGCG plasmids expressing eGFP alone (eGFPonly) or together with the indicated vpu alleles. A construct expressing NL4-3 Vpu was used as a positive control. Click here for file

Additional file 3: Figure S2 Levels of CD4 surface expression levels of 293T cells and HeLa cells transfected with a pCGCG plasmids expressing eGFP alone (vector) or together with CD4 and on TZM-bl cells stably expressing CD4. The numbers give the mean fluorescence intensity of CD4 expression by the transfected eGFP + cell population. Click here for file

Additional file 4: Figure S3 Interaction of wild-type and chimeric HIV-1 and SIVcpz/gor Vpu proteins with ß-TrCP and CD4. Interaction of Vpu with (A) ß-TrCP and (B, C) CD4 in the (B) absence and (C) presence of a dominant negative mutant of β-TrCP1. Refer to the legend to Figure 6 for further detail. Click here for file
