# Supplementary material for: The transmembrane domain of HIV-1 Vpu is sufficient to confer anti-tetherin activity to SIVcpz and SIVgor Vpu proteins: cytoplasmic determinants of Vpu function
Source: Retrovirology. 2013 Mar 20;10:32. doi: 10.1186/1742-4690-10-32 (PMC3621411; doi:10.1186/1742-4690-10-32)
Supplement: Additional file 1: Table S1 — Origin of HIV-1, SIVcpz and SIVgor vpu alleles analyzed. [file 1742-4690-10-32-S1.docx]

**Supplementary Table 1.** Origin of HIV-1, SIVcpz and SIVgor *vpu* alleles analyzed.

**Clone Group Species/subspecies/origin Vpu (bp) Source Accession**

NL4-3 HIV-1 M human 246 IMC/human T cell line U26942

MB897 SIVcpz*Ptt* Central chimpanzee (*P.t.t*) 249 fecal viral RNA EF535994

EK505 SIVcpz*Ptt* Central chimpanzee (*P.t.t*) 240 fecal viral RNA DQ373065

TAN3 SIVcpz*Pts* Central chimpanzee (*P.t.s*) 252 fecal viral RNA DQ374658

ANT SIVcpz*Pts* Central chimpanzee (*P.t.s*) 252 human PBMC culture U42720

CP2139 SIVgor Western lowland gorilla (*G.g.g*.) 240 fecal viral RNA FJ424866

BQ664 SIVgor Western lowland gorilla (*G.g.g*.) 240 fecal viral RNA FJ424867

*P.t.t., Pan troglodytes troglodytes; P.t.s., Pan troglodytes schweinfurthii, G.g.g., Gorilla gorilla gorilla;* IMC, infectious molecular clone.
