# Supplementary figures and images for: The transmembrane domain of HIV-1 Vpu is sufficient to confer anti-tetherin activity to SIVcpz and SIVgor Vpu proteins: cytoplasmic determinants of Vpu function
Source: Retrovirology. 2013 Mar 20;10:32. doi: 10.1186/1742-4690-10-32 (PMC3621411; doi:10.1186/1742-4690-10-32)

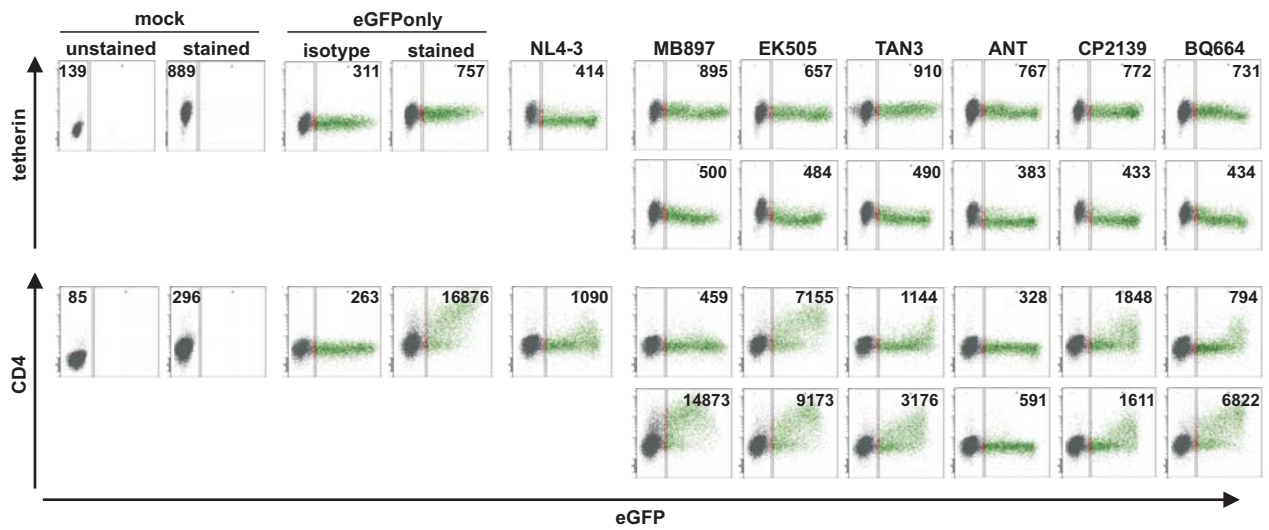

Supplement: Additional file 2: Figure S1 — Tetherin (upper panel) or CD4 (lower panel) surface expression levels of HeLa cells co-transfected with a CD4 (lower panel) expression vector and pCGCG plasmids expressing eGFP alone (eGFPonly) or together with the indicated vpu alleles. A construct expressing NL4-3 Vpu was used as a positive control. [file 1742-4690-10-32-S2.pdf]

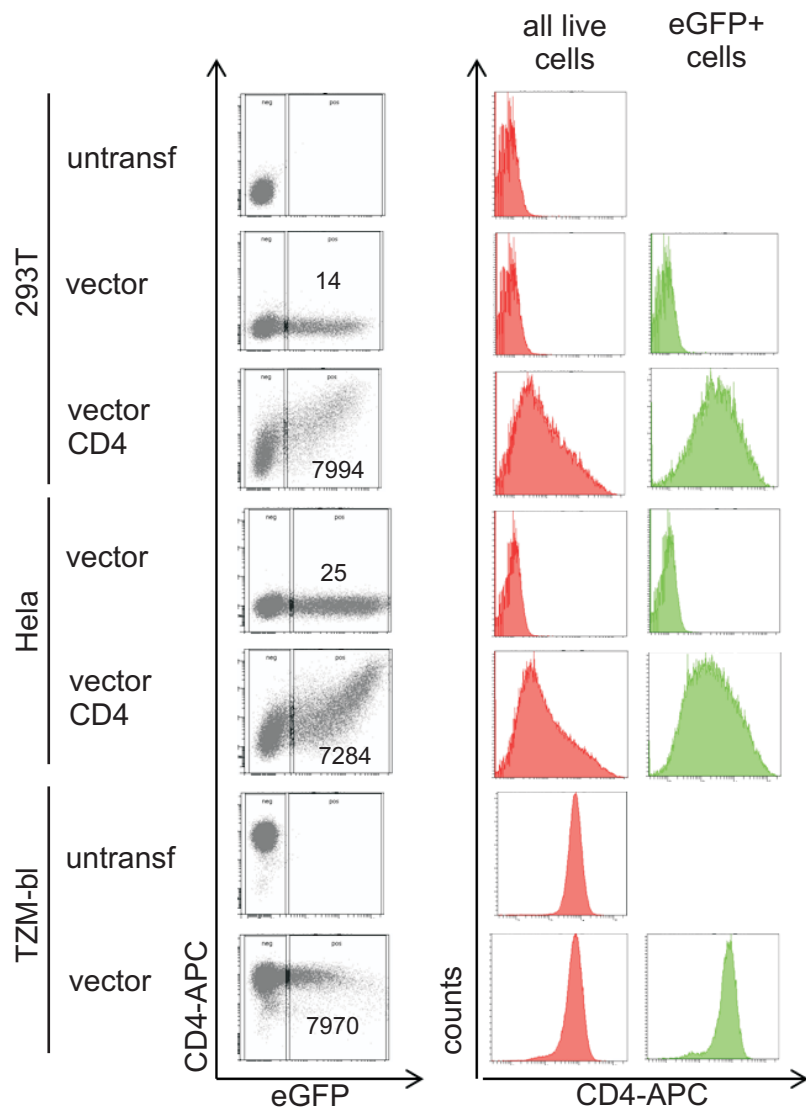

Supplement: Additional file 3: Figure S2 — Levels of CD4 surface expression levels of 293T cells and HeLa cells transfected with a pCGCG plasmids expressing eGFP alone (vector) or together with CD4 and on TZM-bl cells stably expressing CD4. The numbers give the mean fluorescence intensity of CD4 expression by the transfected eGFP + cell population. [file 1742-4690-10-32-S3.pdf]

**A**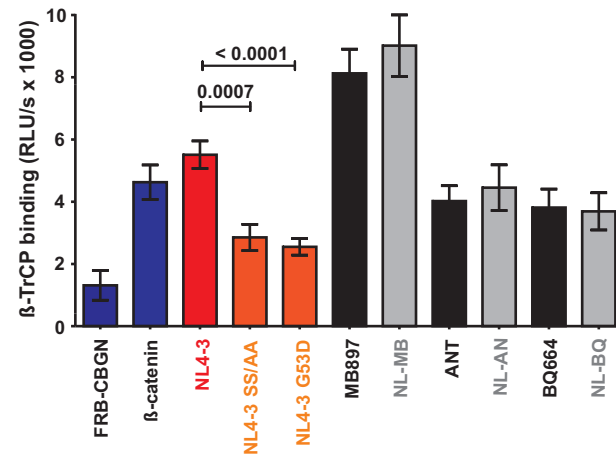**B**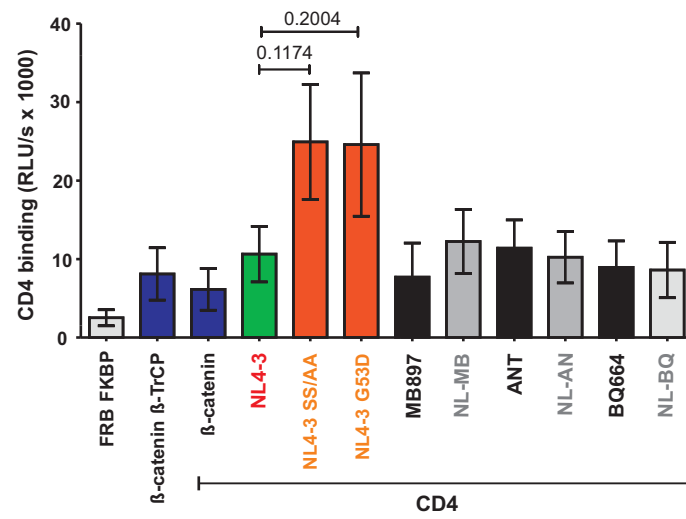**C**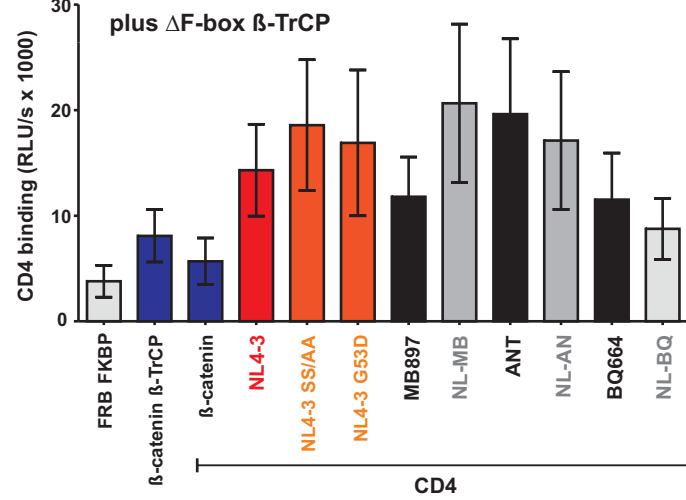

Supplement: Additional file 4: Figure S3 — Interaction of wild-type and chimeric HIV-1 and SIVcpz/gor Vpu proteins with ß-TrCP and CD4. Interaction of Vpu with (A) ß-TrCP and (B, C) CD4 in the (B) absence and (C) presence of a dominant negative mutant of β-TrCP1. Refer to the legend to Figure 6 for further detail. [file 1742-4690-10-32-S4.pdf]
